# FACILITATORS OF PHYSICAL ACTIVITY IN OLDER ADULTS AND THE IMPACT OF FEAR OF FALLING: WHAT KEEPS US MOVING?

**DOI:** 10.1093/geroni/igad104.3022

**Published:** 2023-12-21

**Authors:** Bryant O’Leary, Justin Whitten, David Graham, Dawn Tarabochia

**Affiliations:** Montana State University, Missoula, Montana, United States; Montana State University, Missoula, Montana, United States; Montana State University, Missoula, Montana, United States; Montana State University, Missoula, Montana, United States

## Abstract

Habitual participation in physical activity provides many well-documented health benefits for older adults. It has been suggested that high self-efficacy and internal motivation are facilitators of sustained participation, however, the findings are equivocal. Moreover, research suggests that fear of falling may negatively impact self-efficacy, leading to diminished physical activity levels. Therefore, the purpose of this study was to identify factors associated with sustained participation in physical activity and the potential influence of fear of falling on participation in physical activity for older adults. A phenomenological study was conducted with a convenience sample of 19 community-dwelling older adults aged 55-85. Participants completed an online, open-ended survey to identify potential facilitators influencing habitual physical activity in older adults. Van Manen’s (1990) hermeneutical approach was employed to analyze data. Transcripts were analyzed for themes by two members of the research team. Trustworthiness was established by a peer reviewer who assessed theme accuracy. Self-motivation and life-long participation were identified as facilitators of sustained participation in physical activity. This study supports internal motivation as a facilitator; self-efficacy was not identified. Instead, lifelong participation was identified. For this study lifelong participation was defined as participating in physical activity from childhood throughout adulthood and into older adulthood. Furthermore, modification of habitual physical activity was reported due to fear of falling, namely those associated with outdoor activities. All other reported forms of physical activity were maintained. The influence of these modifications on sustained participation in physical activity for older adults is unclear and warrants further investigation.

